# Optic nerve sheath diameter in critically ill patients: nuances and interpretation

**DOI:** 10.1186/s13054-020-03084-1

**Published:** 2020-06-15

**Authors:** Amos Lal, Kamal Kant Sahu, Ajay Kumar Mishra, Jamal Akhtar

**Affiliations:** 1 Department of Medicine, Division of Pulmonary and Critical Care Medicine, Mayo Clinic, Rochester, MN USA; 2 Department of Medicine, Saint Vincent Hospital, Worcester, MA USA; 3 Department of Sleep Medicine, Montefiore Medical Center, Bronx, New York, NY USA

To the Editor,

We read with great attention the remarkable research letter by Yang and colleagues studying the utility of optic nerve sheath diameter (ONSD) in predicting sepsis-associated encephalopathy (SAE) (surrogate for raised intracranial pressure) in critically ill patients [1]. However, careful reading of the letter raises some concerns that could alter the interpretation of results and overall conclusion. It has been well known that the use of corticosteroids and/or sudden discontinuation can significantly increase the risk of intracranial hypertension and thereby alter the dimensions of the ONSD [2–4]. It has been hypothesized that the sudden withdrawal of corticosteroids can reduce the absorption of cerebrospinal fluid (CSF) and can cause increased resistance to the flow of CSF thereby resulting in intracranial hypertension [4]. Prolonged use of corticosteroids can also create a biochemical scenario similar to vitamin A toxicity with increased carotene levels [2]. This may in turn behave like pseudotumor cerebri and thus intracranial hypertension. It is important to know since many of the critically ill patients in the intensive care unit (ICU) require steroids for a plethora of reasons such as stress dose steroids for septic shock. Sepsis itself has been identified as a clinical state with deficiency of intrinsic corticosteroids. Authors while describing the baseline characteristics of patients should shed more light on this, if these patients in all 3 groups were matched in terms of steroid use.

Systemic hypertension itself could be an independent risk factor for increase in ONSD and has been studied in the past [5]. While presenting the baseline characteristics of the 3 groups studied by the authors, they have omitted the important details specifically about the percentage of hypertensive patients (and baseline blood pressure values at the time of assessment of ONSD).

We wish to congratulate the authors for this remarkable work in the critical care setting to risk stratify patients with SAE with the help of ONSD. However, providing further details about the clinical status of the patients in this setting (such as blood pressure readings) and details of past medical background (steroid use and history of endocrinopathies) will provide readers of this journal with much needed clarity and further credibility to this outstanding work.

## Data Availability

Not applicable.
